# Clinical applications of Genome Polymorphism Scans

**DOI:** 10.1186/1745-6150-1-16

**Published:** 2006-06-06

**Authors:** James L Weber

**Affiliations:** 1 Center for Human Genetics, Marshfield Clinic Research Foundation, Marshfield, WI, USA; 2 PreventionGenetics, 3700 Downwind Drive, Marshfield, WI 54449, USA

## Abstract

Applications of Genome Polymorphism Scans range from the relatively simple such as gender determination and confirmation of biological relationships, to the relatively complex such as determination of autozygosity and propagation of genetic information throughout pedigrees. Unlike nearly all other clinical DNA tests, the Scan is a universal test – it covers all people and all genes. In balance, I argue that the Genome Polymorphism Scan is the most powerful, affordable clinical DNA test available today.

**Reviewers**: This article was reviewed by Scott Weiss (nominated by Neil Smalheiser), Roberta Pagon (nominated by Jerzy Jurka) and Val Sheffield (nominated by Neil Smalheiser).

## Open peer review

Reviewed by Scott Weiss (nominated by Neil Smalheiser), Roberta Pagon (nominated by Jerzy Jurka) and Val Sheffield (nominated by Neil Smalheiser). For the full reviews, please go to the Reviewers' comments section.

## Introduction

Already today, and much more so in the future, DNA sequence information will be used to establish and confirm diagnoses, to help determine treatment, and to prevent disease through presymptomatic identification of genetic risk. Many distinguished authors have recently elaborated upon these points [1-3]. In this article, I describe the many clinical applications of Genome Polymorphism Scans along with some technical aspects of the Scans and limitations in their use.

Genome Polymorphism Scans (hereafter Scans) are defined as the typing of a set of DNA polymorphisms spanning the length of each chromosome within a genome. The Scans are usually performed on individual DNA samples, but occasionally also on pools of DNAs. The two key parameters of the Scans are the type(s) of polymorphisms utilized and the density of polymorphisms. In addition to the actual genotypes, other important data can and should be collected during the Scans (Table 1). Genotyping methods that allow the collection of all these data are preferable.

**Table 1:** Data that may be collected for each genotype in a Scan

1. The identities of the alleles (as complete as possible) at each locus
2. The confidence in the allele calls
3. The detection signals for each allele and whether these signals are normal or are unexpectedly strong or weak
4. The (rare) presence of three or more alleles for multiallelic polymorphisms
5. The expected frequency of the observed genotype given known allele frequencies for the individual's population
6. When genotypes are available from family members, whether the allele transmission patterns between generations are consistent with Mendel's rules

## Applications of Genome Polymorphism Scans

The many clinical applications of the Scans are described in the following paragraphs and listed in Table 2. The applications were developed through the work of many different investigators as well as through the performance in my lab of Scans on over 100,000 people. The applications are listed approximately in the order of lowest to highest numbers of polymorphisms required.

**Table 2:** Minimum STRP^a ^densities required for Scan applications

APPLICATION	MINIMUM NUMBERS OF STRPS
Gender determination	10
Family tree construction	10
Individual identification	10
Chimerism discovery	100
Aneusomy detection	500
Uniparental disomy detection	500
Autozygosity determination	500
Inbreeding measurement	500
Linkage mapping	500
Geoancestry estimation	500
Haplotype determination	500
Propagation of genetic information	1000
Genotyping error detection	1000
Association mapping	2000

^a ^A rough estimate of the equivalent numbers of diallelic polymorphisms can be obtained by multiplying the numbers of STRPs by 4.

A simple, but important, application is the determination of gender. This is achieved through the typing of polymorphisms on the sex chromosomes. Males with normal karyotype, for example, should not be heterozygous for polymorphisms exclusive to the X chromosome, and normal females should show no signals for polymorphisms exclusive to the Y chromosome. Confirmation of gender is one good way to monitor sample mislabeling and rates of genotyping error.

The Scans also permit detection and confirmation of close family relationships. Monozygotic twin, parent-child, full sibling, and half sibling relationships can usually be clearly distinguished from each other and from other relationships with Scans of even modest polymorphism density [4-6]. Pairs of more distantly related individuals can often be distinguished from unrelated pairs, but the exact nature of the relationship is difficult to determine. Comparison of relationships derived from the Scans with patient self-reported family trees should nearly always result in accurate pedigrees. Confirmation of reported pedigree structure is another good way to check for sample mislabeling, and is vital for the confident use of pedigrees in further analyses.

Even very low density versions of the Scans can be used to "fingerprint" individuals [7,8]. Such individual tags have important application in criminal investigations and identification of remains. Some people are already beginning to advocate DNA fingerprinting of all individuals [9]. A key question with identity testing is whether the data generated for clinical purposes should be available to governments and/or law enforcement organizations. Similarly, we need to decide whether different polymorphisms should be used for clinical Scans and forensic fingerprints.

In principle, Scan data can also be used to detect chimeric and mosaic individuals. Much depends on the ability of the genotyping method to detect weak second or third alleles. Such alleles may arise from somatic mutation, sharing of cells between dizygotic twins, fetal-maternal cell transfer, and a number of other mechanisms [10-12]. The presence of foreign cells may cause autoimmune and other health problems [13,14]. It is difficult to distinguish laboratory contamination of a DNA sample from true chimerism or mosaicism. In some cases Scan data from close family members would help to resolve the two possibilities. This application of Scan data is probably the least well established of all those described in this article.

Genome Polymorphism Scans can be used to identify chromosomal or segmental aneusomies. Three or more copies of a chromosomal segment in an individual may often be detected as genotypes with more than two alleles or as heterozygous genotypes with unequal allele detection signals. A good example is the duplication on chromosome 17p that is responsible for Charcot Marie Tooth disease type 1A [15]. A single copy of a chromosomal segment may be identified in at least three ways: as an unusually weak allele signal compared to sequences on other chromosomes or compared to the same polymorphism in other individuals [16], improbably long stretches of adjacent polymorphisms that all appear to be homozygous [17-19], and/or Mendelian parent to child transmission inconsistencies [20].

Chromosomal translocations and inversions may at least occasionally be detected through the Scans. For translocations, if family size is sufficiently large, expected linkage between adjacent polymorphisms that have been separated by the translocation may be diminished or absent. For inversions, highly improbably tight double recombination events may be observed [21], or detection may be achieved through association with specific alleles [22].

Uniparental disomy may be detected through Mendelian transmission errors or, in the case of isodisomy, through improbably long chromosomal stretches of homozygous genotypes [23]. A particularly nice example was involved in the discovery of mutations in the lamin A gene as the cause of Hutchinson-Gilford progeria [24]. Since all cases of this disease are caused by *de novo *mutations, the lamin A gene could not have been mapped by linkage; it was only mapped through observation of uniparental isodisomy on chromosome 1q.

Homozygosity of a polymorphism, especially homozygosity for a rare allele or a contiguous chromosomal stretch of homozygous polymorphisms may indicate autozygosity. Autozygosity is the inheritance of the same chromosomal segment, originally from a more distant ancestor, through both mother and father [25]. Because of population bottlenecks and resulting relatively low genetic diversity in humans, autozygosity for short chromosomal segments is found in everyone. Surprisingly, long autozygous segments spanning up to many tens of mb are also relatively common [26]. Autozygous regions are important clinically because many disease risk alleles have much more potent effects when present in two copies than when present in only one copy [27-30]. When Scan data from parents is unavailable, cytogenetics or comparative genomic hybridization might be required to distinguish a deletion from autozygosity or uniparental isodisomy.

Inbreeding, at least at higher levels, can be detected through Scans [31]. Inbreeding levels in the offspring of prospective couples can also be estimated through Scan data. Even modest levels of inbreeding may have substantial effects on health [32,33].

For many Mendelian disorders, mutations within any of two or more unlinked genes can cause disease. A good example is early onset breast/ovarian cancer where mutations in two genes, *BRCA1 *on chromosome 17 and *BRCA2 *on chromosome 13, are causative [34,35]. If Scans have been performed on family members, then it will often be possible through standard linkage analysis to determine which gene is involved in a particular family. This will substantially reduce testing costs by allowing labs to focus on the correct gene. For this purpose, families do not have to be large enough to obtain lod scores above 3.0, but rather just large enough to indicate which gene likely carries the mutation. In many cases, only two affected family members will be sufficient. In the case of rare recessive disorders in isolated populations, a single affected individual will often be sufficient.

The Scans also allow approximate determination of geoancestry [36,37]. By geoancestry, I mean the geographical origin of a person's ancestors at about the time of Columbus (~1500). An example is presented in Table 3. Beyond personal curiosity, geoancestry is important in cataloging which disease risk alleles an individual may carry. For example, a person of only Northern European ancestry is unlikely to carry the sickle cell anemia mutation, and a person of only African ancestry is unlikely to carry the *CFTR *ΔF508 mutation. However, an African American with 30% European ancestry has a much higher probability of carrying this *CFTR *mutation. As polymorphism density in the Scans increases and as our ability to analyze Scan data for geoancestry improves, it will become possible to confidently determine not only overall geoancestry, but also geoancestry of individual chromosomal segments [38].

**Table 3:** Geoancesty^a ^of selected research subjects from the Dominican Republic

Subject	Caucasian	African	Native American	Asian
A	.02	.95	.02	.01
B	.91	.01	.08	.00
C	.01	.00	.99	.00
D	.89	.08	.02	.01
E	.28	.68	.04	.00
F	.42	.45	.13	.00
G	.17	.46	.36	.01

^a ^Subjects underwent 400 STRP Genome Polymorphism Scans [63]. Geoancestry was determined using STRUCTURE [64] and data from the Human Diversity Panel [65].

Another simple, but very important consequence of performing Scans in families is that haplotypes will often be determined unambiguously. When both parents and a child are typed, the phase in the child can be obtained for all situations except when all three individuals are heterozygous with the same genotype. Haplotypes are more useful than genotypes in many clinical situations, like for example, in the prediction through association of specific mutations that a patient is likely to carry [[39] and see below].

The Scans permit propagation of genetic information through families. Once a single family member is identified as carrying a disease risk allele on a particular haplotypic background, then other family members who carry the risk allele may be identified as those who carry this same haplotype [40,41]. When disease risk alleles are relatively common in a population, then screening all individuals may be cost effective, but when the risk allele is rare, then it is impractical to screen everyone [42,43]. When several different genes are responsible for a disorder and/or when disease genes have many exons, it is expensive to identify the causative mutation. It is unconscionable to repeat this costly analysis for each family member. The presence of strong positive interference in humans [44] makes the propagation process more reliable because double recombination events within small genetic intervals (roughly ≤5 cM) are extremely rare.

**Figure 1:**
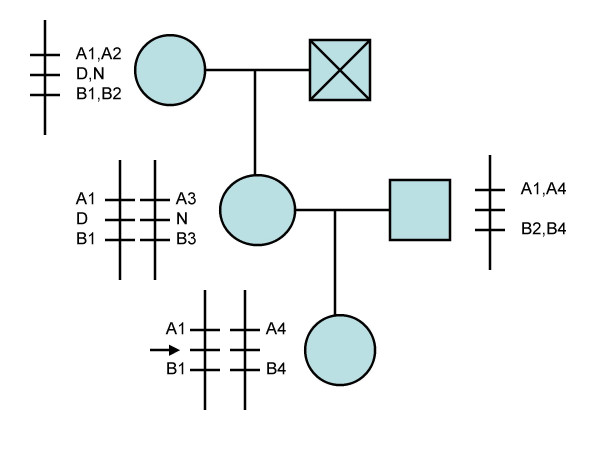
**Hypothetical example of detection of genotyping errors through Genome Polymorphism Scans**. Shown are two multiallelic polymorphisms 5 cM apart from a Scan, A and B, which flank a disease locus (indicated by the arrow in the granddaughter) with a rare disease allele D and normal allele N. Unambiguous haplotypes are determined by inspection in both mother and granddaughter. Barring a highly improbable event, the granddaughter will carry the D allele on the haplotype inherited from her mother. Any test result in the granddaughter at the disease locus which does not yield a D, N genotype is very likely incorrect and should be repeated.

A corollary of the propagation of sequence information through kindreds, is the identification (and hence elimination) of genotyping errors [45]. A simple example is shown in figure 1. Each living family member has undergone the Scan. Multiallelic polymorphisms A and B from the Scan are 5 cM apart and flank a disease gene with rare disease allele D and normal allele N. Typing of the disease locus in the grandmother and mother establishes the haplotypes in the mother. Assuming that the Scan genotypes are correct, and barring highly improbable mutation, gene conversion, or double recombination, then the granddaughter must have inherited the haplotypes shown and must carry the D allele. If genotyping at the disease locus in the daughter yields N, N, then an error is very likely and the test should be repeated.

Finally, through association (linkage disequilibrium) the Scans may be used to suggest which specific mutations a patient is likely to carry. This strategy has been firmly established by the finding that many (probably the great majority) of mutations responsible for disease have arisen from a single founder on a single haplotypic background (as opposed to recurrent mutations at a mutation hotspot) [46-48]. Detection of this haplotype through the Scans will often define the exact mutation in an affected individual and will often predict the presence of a specific mutation in an unaffected carrier. For recent mutations, such disequilibrium may extend several mb. For older mutations, higher polymorphism densities in the Scans will be required.

## Markers used in Genome Polymorphism Scans

Considering abundance and genotyping cost, there are today only two possible choices for polymorphisms to be used in the Scans: multiallelic short tandem repeat (STR; also called microsatellite) polymorphisms or diallelic polymorphisms (either substitutions (SNPs) and/or indels). A comparison of the properties of the two types of polymorphisms is shown in Table 4. Diallelic polymorphisms have the advantages of generally lower genotyping costs and greater abundance. Multiallelic polymorphisms have the advantages of higher informativeness, the apparent ability to detect linkage disequilibrium at much greater distances [49], and the presence of rare alleles which help in the detection of biological relationships, inbreeding and aneusomies, and which increase haplotypic diversity. Considering all these factors, I currently favor the combined use of both types of polymorphisms in the Scans. Others have demonstrated the usefulness of combinations of both types [50-52].

**Table 4:** Average properties of human multiallelic and diallelic polymorphisms

PROPERTY	MULTIALLELIC	DIALLELIC
Number in human gene pool	10^6^	12 × 10^6^
Spacing	3000 bp	250 bp
Heterozygosity^a^	75%	30%
Rare alleles^a^	Yes	No

^a^ For the markers that would typically be used in Genome  Polymorphism Scans.

Although some applications of the Scans, like for example individual identification, can be achieved with quite small numbers of polymorphisms (Table 2), higher polymorphism density is always preferable. Higher polymorphism densities yield greater power for all applications. More distant biological relationships can be established, lower levels of inbreeding can be reliably measured, and shorter duplications and deletions can be detected. Detection of linkage disequilibrium is especially sensitive to polymorphism density.

Polymorphism density in the Scans will almost certainly be limited by cost. As seen by the data in Table 2, the minimum effective polymorphism density for clinical applications would probably be about 1STRP per 4 cM (~1000 STRPs total). The fraction of health care spending that people will be willing to devote to clinical genetic testing is uncertain. Assuming it is 1% or about $60 per year per person, then I believe that the total costs of the Scans should be no more than a few hundred dollars. Despite the crude nature of these estimates, it is important to note that 4 cM density is readily achievable for a few hundred dollars even at today's genotyping costs. As genotyping technology improves, much higher densities should become possible.

A few other factors may affect polymorphism choice and density. For example, several have suggested that local polymorphism density in the Scans should parallel gene density [53,54]. Also, as the number of known common disease risk alleles increases, at least many of the polymorphisms in the Scans could be comprised of polymorphisms that would do the double duty of achieving the applications described in this article and at the same time help to outline the patient's risk for specific health problems. Examples are common apolipoprotein E, β-globin, and hemochromatosis polymorphisms. Polymorphisms may also be chosen so as to determine the orientation of large scale chromosomal rearrangements [22]. Finally, as polymorphism density in the Scans increases, it may become important to consider the location of the polymorphisms relative to strong recombination hot spots.

## Limitations and obstacles

Some applications described in this article, for example the propagation of information throughout kindreds and the determination of haplotypes, require the cooperation of family members. The power of these applications is diminished by the absence of DNA from some family members. A public health system in which DNA is routinely collected from all patients, for example at birth, would clearly increase the power of the Scans.

Much new software and many new data management systems will need to be created to make maximal use of the Scan data. Existing software can certainly be used as a starting point, but much theoretical and applied research still remains.

Propagation of genetic information throughout families will not permit the detection of most new mutations. Compared to inherited mutations that increase the risk for disease, new mutations that influence disease are rare, but of course do continuously occur.

Other genome wide tests such as gene expression analysis, cytogenetics and comparative genome hybridization (CGH) can also be considered for widespread application in patients. Of these other tests, CGH is probably the leader. CGH using high density arrays [55-58] permits much higher resolution mapping of aneusomies than Scans, and will likely find wide application in many individuals. It may even be possible to combine polymorphism Scans with copy number determination [59].

What about just sequencing the entire genome of each patient? There has been much recent discussion and research spending directed toward the goal of sequencing an individual's genome for about $1000 [60-62]. If very low cost sequencing were available, it would clearly accomplish nearly all the applications of the Scans and would also permit the detection of new mutations. However, it currently costs roughly $10 million to sequence a person's genome with a relatively high level of completeness and accuracy. It may be many decades before we achieve the "$1000 genome". Also, even when such technology becomes available, some level of sequencing errors will inevitably be present. The Scans might still be useful in the detection of these errors (see figure 1).

Over at least the next few decades, a more realistic scenario than the "$1000 genome" may be technology for partial, but significant, sequencing of a person's genome for say $100,000. If Scan data is available on family members, then this partial sequencing information from one or two family members can be propagated throughout the kindred. Wealthy individuals may decide to pay for such sequencing out of pocket as a gift to their families. The same principle holds for any other high-information, high-cost tests, like for example, use of a 500,000 SNP chip.

## Concluding comments

From the human and other genome projects, we have learned that whole genome approaches are nearly always more efficient than strategies in which portions of the genome are studied independently. This has been demonstrated for genetic and physical mapping as well as genomic sequencing. I argue that it is also time to switch to a genome wide approach for clinical DNA testing. The current clinical genetics approach of separate counseling, DNA collection, and testing for each locus is hopelessly inefficient if our goal is to substantially increase use of genetic information in health care. Universal genetic tests that can be performed in large numbers of patients are vastly more cost efficient than personalized genetic tests.

The Genome Polymorphism Scan certainly qualifies as such a universal test. It covers all genes in all individuals. Despite the clinical promise of comparative genome hybridization, this and other currently affordable genome wide tests do not even come close to the number of applications described in this article. If the Scans were performed on large numbers of patients, then the resulting data would also comprise a vast pool of information that could be mined for research purposes. We have the necessary financial resources and technology. I believe we should begin immediately.

## Abbreviations

Scan Genome Polymorphism Scan

cM centiMorgan

STR short tandem repeat

STRPs short tandem repeat polymorphisms

SNPs single nucleotide (base substitution) polymorphisms

CGH comparative genomic hybridization

## Reviewers' comments

### Reviewer's report 1

Scott T. Weiss, M.D., M.S., Professor of Medicine, Harvard Medical School, Boston, MA, USA

Weber provides a comprehensive review of Genome Polymorphism Scans in his review article. He has extensive experience in this area having run the microsatellite genotyping service for NHLBI for over 10 years and having performed many of these scans. He identifies 14 different potential uses for STRP scans of varying density, and he comprehensively covers the world of scanning from the viewpoint of the genotyper. Despite the wealth of information in the article there are other perspectives that would have provided greater comprehensiveness to this review and greater information for the reader.

For example, who you genotype (ie your study design) is as important as what type of markers and the marker density you use. Do you have a single family? A collection of affected sib pairs? Extended pedigrees? What is the goal of your scan? Do you wish to perform linkage for a complex trait? Map a single gene disorder? Weber doesn't address study design at all for the 14 different types of scans. Nor does he provide the reader with what he would recommend for each of his 14 applications, leaving the novice to wonder about how best to approach each problem. This results in several controversial and potentially confusing points.

For example use of SNPs (diallelic markers) for linkage is still controversial, especially for extended pedigrees, because statistical software to analyze this data is still not really available. Also, for association mapping it is unlikely that 8000 markers (4 × 2000) is really enough to cover the whole genome. It would cover a sizable region for LD mapping of a linkage peak.

Despite these deficiencies the paper distills a wealth of experience with genome scans from an experienced practitioner of the art and presents a comprehensive delineation of its potential uses in genetics.

### Reviewer's report 2

Roberta A. Pagon, M.D, Professor of Pediatrics, University of Washington, and Division of Genetics and Development M2-9 Children's Hospital and Regional Medical Center Seattle, WA, USA

Dr. Weber raises provocative and, I think still futuristic, comments about the use of Genome Polymorphism Scans ("Scans) in *clinical *care. Scans, defined as the typing of a set of DNA polymorphisms spanning the length of each chromosome within a genome, provide a set of information about normal variants in an individual, which Dr. Weber calls a "universal test". Most current *clinical *molecular genetic testing, by his definition, is "personalized genetic testing", i.e., it is focused on identifying in an individual specific disease-causing alleles to (1) establish disease causation or (2) establish disease risk based on family history or race/ethnicity. Research testing for (1) and (2) are totally different issues; as is forensic testing.

One can look at the clinical (not forensic, not research) uses for Scans regarding their ability to accomplish (1) and (2) above.

A strength of the proposed current clinical use of Scans is that linkage disequilibrium can offer a prediction for certain mutations within a gene.

Weaknesses in the proposed current clinical use of Scans are:

In general, in the current social and health payer environment, most testing needs to be done on individuals, not families. The logistics of sample collection on far flung families are problematic and in the US third party payer reimbursement on testing of relatives (not probands) is almost insurmountable.

Scans are limited in the ability to identify disease-causing genetic alterations. They may be able to identify segmental aneusomy (but additional testing is likely to be necessary to interpret the results with certainty). Furthermore, current research with comparative genomic array analysis has identified copy number to be a polymorphism that confounds test result interpretation and has underscored the comment of Dr Weber that the software needs for such analysis are just beginning to be addressed.

Tracking multiple disease risk alleles (for common complex disorders, such as diabetes mellitus, coronary artery disease) in a family has great future potential, but limited current application because the search for these disease risk alleles, the ability to interpret their significance for individuals, and the understanding of dietary/health/environmental interventions that can reduce the risk itself are still in the discovery stage.

The use of Scans in healthcare will require vast amounts of genomic data and phenotype data that are updated as individuals age. These significant issues are beginning to be addressed at the national level by the National Institutes of Health, so there is no doubt that clinical applications of genomic polymorphism scan data will be useful in healthcare, the question is when.

### Reviewer's report 3

Val C. Sheffield, M.D., Ph.D., Professor of Pediatrics, University of Iowa, Iowa City, USA

In this article, Dr. James L. Weber reviews methods and applications of a whole genome polymorphism scan and expresses his opinion that a whole genome scan is the most powerful clinical DNA test available today. Dr. Weber expresses his opinion that a genome scan at a minimum density of one marker every 4 centimorgans is a clinically useful and cost effective test, and he concludes that such a genome-wide polymorphism scan should be applied widely and that "we should begin immediately".

The article is an expansion and follow-up on an article written by Dr. Weber in 1994 entitled "Know Thy Genome". The current article expands upon the previous article by reviewing more in depth current applications of a genome scan. Dr. Weber correctly points out applications of genome-wide polymorphism scans, some of which will be unfamiliar to some readers. These applications include, among others, gender determination, chimerism discovery, aneusomy detection, uniparental disomy detection, autozygosity determination, geoancestry estimation, and disease linkage and association detection. The author is correct that genome-wide scans are a powerful tool and useful for many applications. The author also correctly points out that large-scale genome-wide approaches are more cost effective than small-scale testing. The article is well referenced. The article makes several interesting points, some of which are controversial, and thus will be interesting to the readership.

The article has weaknesses which should be addressed by the author. A major weakness is that the author does not point out that there are major differences between research applications of a genome scan and clinical applications. This weakness is most notable in the section on "Limitations and Obstacles". In this section, the author does not include some of the most significant obstacles to the application of large-scale genome-wide genotyping to clinical care. In his previous article, the author mentioned such obstacles as genetic discrimination and privacy. These issues are not mentioned in the current article. A brief update of where things currently stand with respect to these issues would improve the article.

The author ignores other important issues, and a more balanced recognition of obstacles to applying a genome scan to clinical care would strengthen the article. A few other important obstacles that the author should address to strengthen the article and give a more balanced picture are mentioned below:

Although cost is discussed, the true cost of using a genome scan as a clinical test is not considered. The author makes estimates of the cost of the genome scan and based on these costs describes the cost as affordable. The cost of the actual genotyping is likely affordable. However, clinical applications of the genome scan require sophisticated analyses of polymorphism data and most importantly, proper clinical interpretation of the data. The cost of this interpretation is not considered.

Perhaps the most significant obstacle to the application of a genome scan is the complexity of the data generated by the scan. A genome-wide scan as proposed in the article would contain numerous individual pieces of information, as well as combinations of information that would need to be integrated. The amount of data generated, in fact, is the strength of the scan. But it is also the weakness. Each individual interpretable piece of information generated by the scan would potentially have its own sensitivity and specificity. In many cases, the sensitivity and specificity would be population and/or family specific. Who in the health care system would deliver the proper interpretation to patients and by what means would the information be delivered? It should be noted that currently genetic counseling services are poorly reimbursed by third party payers. The author makes an intriguing comment when he states that "The current clinical genetics approach of separate counseling...is hopelessly inefficient". Discussion of alternative strategies would be of interest.

In recommending widespread application of a genome scan for clinical purposes, the author ignores many of the principles of current screening programs. Two such issues include the availability of a useful intervention (treatment) and the availability of societal infrastructure to inform the patient and family of results, confirm results, and properly implement treatment and counseling. It is of interest that two of the tests mentioned by Dr. Weber as potentially included in the scan are hemochromatosis and apolipoprotein E (*APOE*) genotyping. Hemochromatosis is a treatable disorder, but large-scale screening for this disorder has not been implemented primarily because of issues related to non-penetrance of the disorder, and thus what a positive test means to a given individual. *APOE *genotyping is not generally offered, even though specific alleles are statistically associated with Alzheimer disease and macular degeneration, for several reasons, primarily that there is currently no specific successful intervention for these disorders. The inclusion of this testing in a genome-wide scan would at the present time have potentially negative consequences. Dr. Weber's thoughts on these issues would strengthen the article.

In summary, this article is a review of current applications of genome-wide scans. The author makes interesting and valid arguments regarding the utility of such scans for clinical purposes. The major weakness is that the article does not discuss important obstacles to the clinical application of such a scan. Despite this weakness, I recommend acceptance. The article will help generate important dialogue regarding the wide-spread application of clinical genetic testing. By stating that "we should begin immediately", Dr. Weber has challenged the medical and scientific community to intensity the effort to use genomic data for patient care; the science education community to better educate the public concerning the meaning of genetic information; and each individual to be more involved and responsible for their own health.

### Author's response

*I am grateful to the distinguished Reviewers for their thoughtful comments. Nearly all of the Reviewers' concerns deal not with the genetic and technical issues that are the primary focus of this article, but rather with the practical difficulties involved in introducing the Scans into our health care system. I originally planned to include my thoughts about the future of clinical genetics in this review article, but it seemed that the article was becoming too long. I decided therefore that it would be better to split the material into two manuscripts: this review article dealing with the genetic applications and technical issues of the Scans, and a second perspective article dealing with what I believe should be some of the next major steps in clinical genetics, including of course introduction of the Scans. The second manuscript is in preparation. All of the concerns raised by the Reviewers will be addressed in the second manuscript. At this point, I'll just state that although I agree completely with the Reviewers that there are substantial obstacles to the introduction of the Scans into clinical practice, I also feel that the obstacles are definitely surmountable, and that the time to begin working on these problems is now*.

*Dr. Sheffield argues that some DNA analysis like HFE (hemochromatosis) and APOE (Alzheimer Disease) testing is potentially harmful. While I acknowledge that genetic discrimination and overinterpretation of testing results are potentially significant problems, I also respectfully submit that the basic limitation of clinical genetics today is not too much knowledge of patient's genomes, but rather too little. I believe that a major objective of 21*^st ^*century health care should be to determine the complete or near complete genomic sequence of virtually every patient. This is, of course, the primary rationale behind all the money and efforts that are currently being devoted to achieving the "$1,000 genome"*.

*Finally, Dr. Weiss makes the valid point that low marker density Scans will have quite limited power to detect association. Even at low marker density, however, the Scans will be able to detect association for some genes that are close to the markers, particularly in isolated populations. Hopefully, genotyping technology and marker density will eventually improve to the point that association with nearly all genes becomes practical*.

## Declaration of competing interests

While this article was mostly written while the author was employed at the public Marshfield Clinic Research Foundation, the author is now employed as founder and president of PreventionGenetics, a private DNA banking and testing company.
